# Developing the ‘Life Threads’ approach to support families after traumatic brain injury in UK community settings: protocol for a qualitative prefeasibility study

**DOI:** 10.1136/bmjopen-2024-084204

**Published:** 2024-10-17

**Authors:** Charlotte Jane Whiffin, Caroline Ellis-Hill, Alyson Norman, Morag Lee, Parmjeet Kaur Singh, Jo Clark-Wilson, Audrey Daisley, Natasha Yasmin Felles, Mark Holloway, Sara Rose, Fergus Gracey

**Affiliations:** 1 College of Health, Psychology and Social Care, University of Derby, Derby, UK; 2 Division of Clinical Neurosciences, University of Cambridge, Cambridge, UK; 3 Faculty of Health and Social Sciences, Bournemouth University, Poole, UK; 4 Faculty of Health and Human Sciences, School of Psychology, University of Plymouth, Plymouth, UK; 5 Patient and Public Involvement Representative, Derby, UK; 6 Head First (Assessment, Rehabilitation & Case Management) Limited, Hawkhurst, UK; 7 Connect Neuropsychology, Oxford, UK; 8 Faculty of Medicine and Health Sciences, University of East Anglia, Norwich, UK

**Keywords:** qualitative research, psychosocial intervention, family, brain injuries, feasibility studies

## Abstract

**Introduction:**

Traumatic brain injury (TBI) brings about inevitable and significant changes for family members. Mental health effects for family members are well documented but there are significant gaps in support options across services. Here, we describe our protocol for a study that seeks to investigate the potential for a narrative, creative approach, the ‘Life Threads’ approach, as a low intensity, accessible means of support that can be applied across service contexts.

**Methods:**

We will recruit 20 family members of someone who sustained a TBI from services in an area of the UK with a diverse demographic. Family members will be provided with the ‘Life Threads’ approach materials and guided in their use. The study is planned to run from March 2023 to July 2024.

**Analysis:**

Collection of data on potential usefulness, feasibility and acceptability will be through focus groups and individual interviews. Transcripts will be analysed using reflexive thematic analysis, conducted within a relativist, constructivist orientation.

**Ethics and dissemination:**

Ethical approvals have been sought and gained (Nottingham 1 Research Ethics Committee, 23/EM/0185, 4 September 2023), and the study has been registered with ISRCTN. As a research team, we are guided by our own personal, professional and research experiences of TBI which we will consider reflexively throughout the research process. Results will be disseminated in collaboration with our patient and public involvement group through open peer-reviewed journal publication and scientific conference, with lay summaries shared via recruitment sites.

**Trial registration number:**

ISRCTN17392794.

**STRENGTHS AND LIMITATIONS OF THIS STUDY**

- This study will employ systematic qualitative methods designed to yield interpretable data regarding the ‘Life Threads’ approach.
- The methodology will actively engage with issues of diversity and health inequality to gather diverse experiences of families post-traumatic brain injury (TBI) and with the intervention.
- The study is underpinned by patient and public involvement in the development of the approach and throughout the study, and the research team brings wider personal and professional experience of family experiences post-TBI, ensuring the approach resonates with families’ needs and experiences.
- We are seeking diversity in our sample but there will still be limits to the transferability of initial findings across other contexts.
- Given the nature of the sample, and in-depth examination of individual experiences, it may be challenging to achieve clear and consistent views on intervention design and evaluation to inform a future feasibility randomised controlled trial.

## Introduction

 Traumatic brain injury (TBI) is the leading cause of disability among individuals under 40 years of age in the UK.^1^,^3^ Recovery is commonly incomplete and those who survive are often left with a complex range of physical, psychological, cognitive, behavioural and emotional deficits. Survivors do not often return to their preinjury life without consequence and families are told why their lives and relationships may change.^4^ Family members face many challenges and can experience symptoms of depression, anxiety, stress and reduced life satisfaction^5^,^9^ as a consequence. For example, evidence indicates that a TBI has a significant negative effect on family relationships, lifestyles and quality of life.^10^ Brain injury can also damage the stability of the family system and negatively affect family functioning. Poor family functioning in turn has been associated with emotional distress including anxiety, depression and increased strain^11^,^15^ and is also linked to poorer outcomes for the injured person.^15^ Several studies show that it is not the physical demands of caring that cause the greatest burden but trying to live with changes in personality, behaviour and cognition.^911 16^,^21^ The impact of TBI on the family can, therefore, be seen as inevitable and enduring, and there is increasing recognition that family members are changing post-TBI in response to a major life event^22^ with impacts on mental health and quality of life.^23 24^ There is growing evidence based on formal psychological support or interventions for family members (eg, Soendegarrd *et al*,^25^ Kreutzer *et al*^26^ and Bushnik *et al*^27^) which indicates potential for benefits. Such approaches include a mix of psychoeducation, coping skills and support for adaptation. However, they are typically implemented by skilled therapists or clinical neuropsychologists with specialist knowledge of the effects of brain injury which could limit accessibility. Qualitative research examining the challenges and support among family members of people with ABI showed support for families is inconsistent, and what is available often inadequate for their needs.^28^ In a more recent scoping review, Norman *et al*^29^ concluded ‘those with acquired brain injuries, and their families, experience significant difficulties interacting with community-based services and often do not receive appropriate access’.

Studies have consistently shown the importance of understanding the subjective changes experienced by family members post-TBI.^2830^,^34^ Refined understanding of such changes might help to identify novel ways to support adaptation to life postinjury with less reliance on specialist staff or services. In response to growth in evidence of subjective changes we (CJW, FG and CE-H) conducted a meta-synthesis of thirty qualitative studies (participant N=845) that examined the family experience of living with an adult who has a TBI.^35^ We identified four dimensions of subjective experience and meaning, each of which had two inter-related aspects: displacing and anchoring; rupturing and stabilising; isolating and connecting; harming and healing. The interpretation of these dimensions and aspects revealed the substantial existential work involved for families in negotiating, maintaining family system equilibrium and moving forward in their lives. We concluded that family members have their own unique subjective needs, and while there is a basis for families to have hope for positive experiences, there is often a focus in research on negative experiences. We recommended that further research be carried out that explored the conditions which maximise opportunities to develop richer personal accounts postinjury. This echoes recent developments in the application of narrative approaches such as ‘Tree of Life’ groupwork in neurorehabilitation settings (eg, Mwale *et al*^36^ and Butera‐Prinzi *et al*^37^) and arts-based embodied approaches.^38^ Such approaches contrast with conceptualising needs purely in mental health diagnostic terms (such as ‘depression’) or negative outcomes (such as ‘burden’). While the sample size aids confidence in transferability of the review findings, we noted under-representation of male family members and siblings, and lack of geographical, social and ethnic diversity in the field. We also recommended that an approach focused on family adaptation through supported sense-making with uninjured family members, as an emerging area, warranted further evaluation.

The ‘Life Threads’ model provides a potential basis for such support following brain injury. The model was developed through qualitative analyses of the lived experience of people poststroke and their family members.^39 40^ Drawing on the concept of ‘biographical disruption’ in the face of chronic illness^41^ the original ‘Life Threads’ model conceptualises adaptation to the significant changes arising following stroke in terms of disruption to ‘narrative threads’ of identity. The premise of the model is that humans create a coherent sense of self and sense of control and predictability in an unpredictable world using life stories (or ‘threads’) which connect the past, present and future leading to a state of existential equilibrium. These stories are constantly created and recreated in relationship with self and others in society. Following an ABI, these threads may become frayed, while some remain intact (ie, being a daughter or father) and others may break (ie, being a manager at work, being a great cook, being a football player). Existential equilibrium is lost, and people lose a sense of who they are and how to move forward in life. Diagrams of the model can be seen in the paper by Ellis-Hill *et al*.^39^

The Life Threads model provides several implications for supporting individuals or families in the rehabilitation setting or beyond. When drawing on these ideas in rehabilitation, the role of the rehabilitation team is to support the person to explore how life threads can be reconnected, developed or safely tied off through physical and discursive interventions. The model suggests that adaptive emotional responses can be supported by endorsing a positive view of self, ‘being’ with somebody as well as ‘doing’ things for them and seeing acquired disability as a time of transition rather than simply of loss. The model was effectively drawn on alongside an arts-based approach to inform an arts and health group to support adaptation to life poststroke.^42^ The arts and health method aimed to provide a safe and unstructured space to foster creativity among participants, which in turn is argued to help with exploring issues of identity change and finding new adaptive narratives.^42^ We further advanced the model by overlaying the narrative dimensions constructed from our qualitative meta-synthesis of the experiences of family members following a TBI.^22^ However, the application of the ‘Life Thread’ model as a clinical tool to help family members with their processes of adaptation in the face of the challenges of TBI has not been empirically investigated.

Accordingly, we drew on this prior work, alongside input from family members of those who experienced a TBI, to create a novel supported method of storytelling: the ‘Life Threads’ approach. While we predict potential benefits for family members participating in this, we do not yet know the best way to deliver the ‘Life Threads’ approach or the extent to which it requires facilitation by support staff. The planned study will provide a qualitative understanding of the ways in which families engage with, and make use of, the materials and the approach. In addition, we hope to gain insights into contextual aspects of people’s experience with the approach across diverse groups and contexts in the UK. Alongside feedback on the methods used, the study will yield rich information regarding adaptation of the approach for future evaluation in a feasibility and acceptability randomised controlled trial (RCT). Specifically, we will be able to revise the guide and materials for implementing the approach and make any adaptations required based on feedback and experiences of participants. The qualitative feedback on any effects will also help orientate us to potential primary outcomes for a feasibility RCT. The aims of a future feasibility RCT here would be to establish parameters for a full trial such as selection of primary outcome measures, recruitment rate and estimated required sample size and acceptability of the intervention and study procedures. Given the potential use of the approach across diverse community settings and groups, future formal evaluations of the approach should also embed a qualitative process evaluation, in keeping with critical realist approaches to trial design.^43^

## Aims and objectives

Research aim:

To understand the clinical potential of storytelling through the ‘Life Threads’ approach and gather the information required to plan a feasibility randomised controlled trial.

Primary objective:

- Explore if family members’ find storytelling through the ‘Life Threads’ approach useful as a strategy to support their individual subjective well-being and adjustment post-TBI.

Secondary objectives:

- Assess uncertainties in relation to the clinical application of ‘Life Threads’ approach including acceptability; adherence and level of facilitation required.
- Identify appropriate methods for a feasibility study, including representative recruitment; choice of primary outcomes; mode of delivery and comparator arm(s).
- Understand how family members use the ‘Life Threads’ approach to understand the impact of TBI on themselves and their families.
- Explore if the four domains of subjective experience post-TBI (displacing and anchoring; rupturing and stabilising; isolating and connecting; harming and healing) are representative of family member experiences.

## Methods and analysis

### Study design

We will conduct a in-depth qualitative study that will allow us to explore the value and acceptability of using the ‘Life Threads’ approach with family members post-TBI. Qualitative research attempts to interpret the meaning people bring to their experiences.^44^ This study is situated within an interpretivist paradigm with a relativist ontology and constructivist epistemology. Working with subjectivity and honouring multiple realities^45^ will facilitate an in-depth and exploratory approach, which is consistent with the narrative orientation of the ‘Life Threads’ approach and the related background qualitative work we have conducted. As a research team, our ontological and epistemological positions are also anchored in our axiology^46^ which reflects our values and orientations towards addressing the well-documented distress of family members and doing so in a way which is socially inclusive. These perspectives are rooted in our own personal and professional experiences of brain injury, including lived experience as a family member, as well as professional experiences across the acute-community pathway, representing psychology/neuropsychology, nursing and occupational therapy, and qualitative research experience. This commitment to qualitative philosophy will strengthen understanding of the perceived benefits of using the ‘Life Threads’ approach from the participant’s perspective, in addition to pursuing a deep and rich understanding of the approach and its potential application.

### Patient and public involvement

Patient and public involvement (PPI) has been the cornerstone of this research and fundamental to its successful application for funding from the National Institute for Health and Care Research (NIHR). The lead researchers have met with family members on several occasions, this engagement is summarised in table 1 in accordance with the Guidance for Reporting Involvement of Patient and Public Involvement 2 Short Form checklist.^47^

**Table 1:** PPI input detailed in accordance with the GRIPP2 Short Form Checklist

Aims	Determine the potential value of the Life Threads TBI project. Understand how the Life Threads materials could be used with participants. Confirm the accessibility of participant-facing documents and plain English summary. Consultation and expert guidance throughout the life cycle of the project. Provide insight into ethical issues such as risks and benefits.
Methods	We met with six family members of people with traumatic brain injury in two focus groups prior to making an application for funding to the NIHR Research for Patient Benefit programme. These group members also reviewed the application prior to submission.Two members of the initial advisory group joined the research team as coapplicants and join monthly project management groups.A new advisory group has been recruited to advise and guide the research team during the life cycle of the study.The PPIE group at Nottingham University Hospitals NHS Trust were consulted to advise on the plain English summary and AN is the lead for PPI on the project to ensure PPI members are well supported.
Results	Family members told us the study was worthwhile and reminded us of how important, and meaningful, it was for them to share their individual story with others.Family members were very clear that they wanted the choice to participate to be their own and not to be that of the injured person.Explicit and detailed feedback was given on the participant recruitment materials, the Plain English summary and the ‘Life Threads’ approach materials.
Discussion and conclusions	Individual interviews were added to the data collection methods.Budget was allocated for day care so that a family member could participate by themselves where they had carer responsibilities.The plain English Summary, participant recruitment/consent materials and the ‘Life Threads’ approach were all revised following engagement and feedback.

GRIPP2Guidance for Reporting Involvement of Patient and Public InvolvementNHSNational Health ServiceNIHRNational Institute for Health and Care ResearchPPIpatient and public involvementPPIEPatient and Public Involvement and EngagementTBItraumatic brain injury

### Setting

The study is based in community settings in the East and West Midlands regions of the UK. This setting includes rural and urban locations and has a diverse socioeconomic and ethnic population. We can, therefore, examine the breadth of applicability and understand diverse family experiences. The study start date is March 2023 and end date (defined as completion of the final data collection period) is July 2024.

### Sample

The study will use a non-random, purposive, maximum variation sample^48^ (similar to quota sampling) whereby we will work collaboratively with gatekeepers to recruit people from a range of cultural backgrounds. Regional data suggest approximately 20%–30% of the East/West Midlands identify as Black, Asian or mixed ethnicity. We are committed to reflecting this diversity within the sample.

An initial sample of 50 will be sought, from which a final sample of 20 participants will be selected using maximum variation sampling to reflect regional diversity. This sample size is within the parameters identified for data saturation within qualitative research.^49 50^ However, saturation is a contentious issue^4551^,^53^ and is not aligned with the proposed analytical framework for this study.^54^ We will, therefore, be guided by the principle of ‘data sufficiency’ by Dey.^55^ Sample diversity coupled with transparent reporting of participant characteristics will help reduce risk of selection bias^56^ and aid transferability or applicability of findings.^57^ In the context of this study, this diverse sample of 20 participants was identified as sufficient to address the aims and objectives of this study and feasible within the resource constraints of the project. We will evaluate the sufficiency and representativeness of the sample size during the data analysis phase.

### Participants

Any family member, or close friend, will be eligible to take part (eg, spouse, partner, sibling, grandparent, significant other) and more than one person per family can take part. We will use an inclusive definition of family as ‘the family is who they say they are’^58^ so that any person who identifies as a member of the injured person’s family, including a close friend, is eligible. The current evidence base regarding the experiences of families post-TBI is formed predominantly by respondents who are white, female and in heterosexual relationships.^29 35^ We will make sustained efforts to recruit from other under-researched groups including male relatives and families with same-sex couples. Inclusion criteria are as follows:

- Identifies as a family member or close friend of a person with any severity TBI, sustained at least 2 years prior, age at injury 18 years or older.
- Known to the injured person before injury.
- Age 16 years or above.
- Able to give informed consent.
- Residing within the East/West Midlands of England.
- Have access to a smartphone, tablet or computer and access to the internet.
- Willing to participate in a group.
- Fluent in English.

Within the regions identified for study recruitment, 6.2% of the East Midlands^59^ and 7.2% of the West Midlands^60^ population speak a language other than English as their main language. Given the sample size, collaborative study methods and exploratory nature of this, it was reasonable to restrict the sample to those who speak English at this time. We will, however, recruit all those with sufficient English to engage with the study methods in a way that yields interpretable results. We may then be able to widen recruitment in a subsequent study.

We are mindful of the risk of distress from study participation; however, we do not want to create unnecessary barriers to participation as negative emotions are common among family members and the study aims to relate to the emotional needs of this population. Therefore, we would only exclude those who feel their circumstances or mental health needs might make participation too distressing or risky for them. These risks will be explored during a preconsent meeting prior to recruitment.

### Procedure

**Figure 1:**
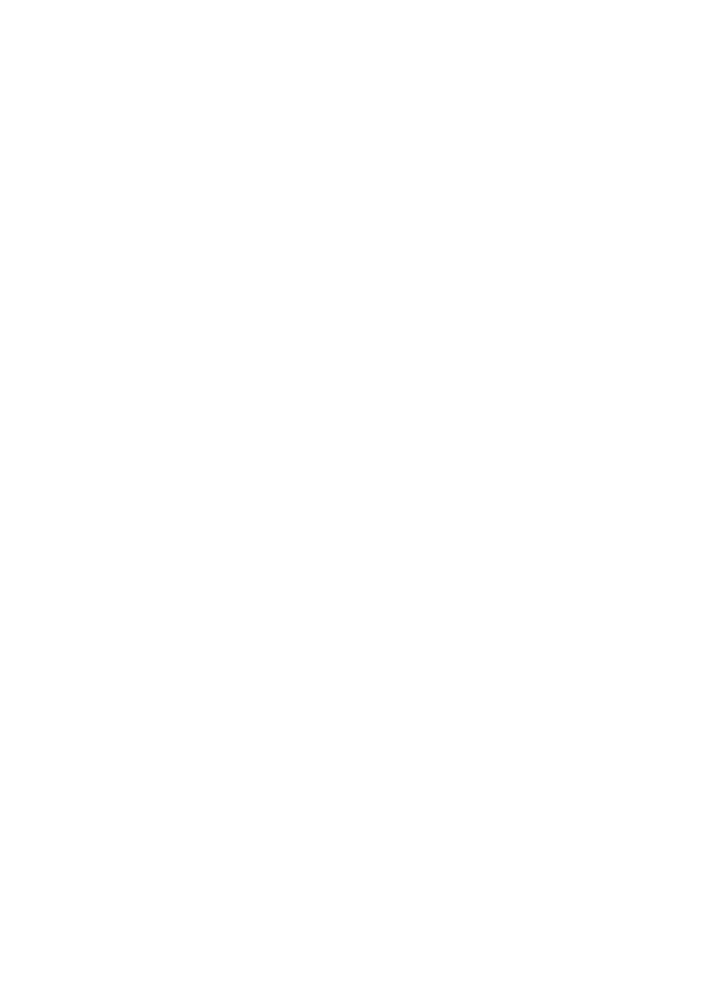
Schematic diagram showing participant flow through the study and key study activities over time. GP, general practitioner; NHS, National Health Service; TBI, traumatic brain injury.

A schematic of participant flow through the study and key tasks is provided in figure 1.

#### Recruitment

We will recruit through three routes: (1) National Health Service (NHS) brain injury services, (2) third sector and (3) social media.

#### NHS brain injury services participant identification sites

A member of the direct care team at three UK NHS sites will review patient records for eligibility and identify the next of kin contact details. The patient will be written to first informing them about the study and notifying them that their family members will be contacted directly by the research team approximately 1 week later. Patients or their supporters may contact the lead researcher if they have any questions. The recorded next of kin will then be written to with a study summary sheet and contact details to express their interest. Depending on local practice preferences the patient, and next of kin, may be given this letter in person during a hospital visit or this letter may be sent in the post/via email.

#### Third-sector participant identification sites

Managers, or equivalent, of regional Headway branches and groups in the East and West Midlands will be given a general notice of study recruitment to be sent to members directly or to be included in newsletters or social media pages. We will also provide a family member letter and study summary sheet to be sent directly to family members who are registered with the local branch/group. This email will be sent by a staff member at the local Headway. Family members can then contact the CI directly to express interest in taking part.

#### Social media

An infographic will be posted on Social Media Channels including Twitter/X, LinkedIn and Facebook. We will tag relevant organisations such as Headway UK, regional Headway groups and branches, the UK Brain Injury Forum (UKABIF, including an affiliated group ‘Anchor Point’ driving change for families after ABI) and an independent brain injury case management company (Head First). We will also ask UKABIF and Anchor Point to act as gatekeepers and send the recruitment details to their mailing list. The infographic has a QR code leading to the study summary sheet providing additional detail prior to contacting the CI to express an interest in the study.

#### Description of ‘Life Threads’ approach

**Figure 2:**
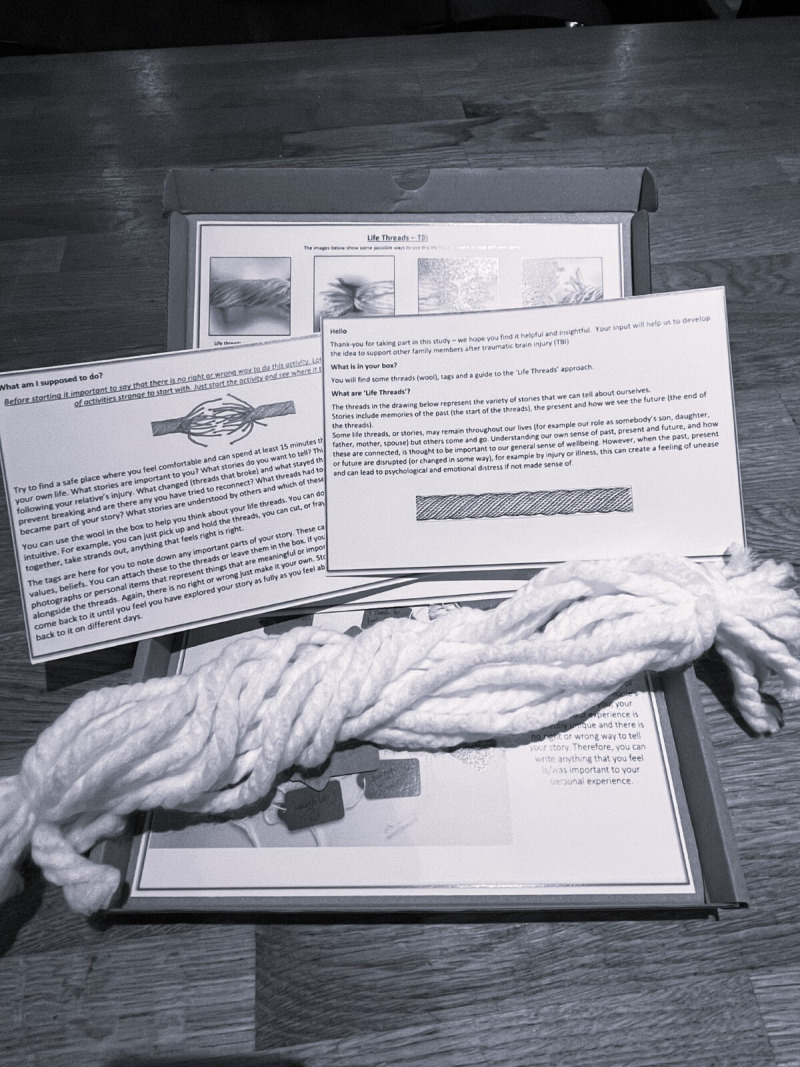
‘Life Threads’ approach materials sent to participants in the post.

The ‘Life Threads’ approach comprises a set of materials and a guide for self-guided or facilitated administration (see figure 2). The practical objective of the approach is for the participant to create a representation of the various intertwined, continuous, broken or ‘frayed’ stories in their life spanning the period before and after their relative’s TBI, and to use this as a vehicle to share their story. The materials include several lengths of wool tied together at each end (denoting the ‘life threads’), tie-on labels of various sizes, instructions on how the materials could be used to represent the person’s various life stories or timelines, and related key events or points in these stories, and pictures illustrating how the materials could be used. In keeping with creative approaches to sense-making in chronic health conditions,^42^ the guide emphasises that there is no right or wrong answer and participants should feel free to use the materials however they choose. In addition to using labels to denote key points in a story, participants are also invited to add photographs or objects that are personally significant and include these in their life threads creations. For facilitated administration, the facilitator (CJW) will support the participant to tell their story using the ‘Life Threads’ approach, providing a curious and non-judgemental response to their creative efforts, encouraging exploration with the materials in whichever way they choose. The way in which the materials are introduced to participants, and data collected on use of materials and the stories thus facilitated is provided below.

### Primary outcome measure

Acceptability and feasibility were measured using data gathered through qualitative interviews and focus groups to remain open to participants’ views and experiences pertinent to the study aims during the study.

#### Data collection

The study is conducted in six stages after informed consent has been provided. Methods were developed in consultation with the research and PPI team but were not formally piloted prior to this study. While participants will be free to withdraw at any stage, it will only be possible to withdraw interview data up to 7 days after completion of the research interview. It will not be possible for participants to withdraw focus group data from analysis although participants will have up to 7 days to request their responses in the focus group (full or partial) are not included in publications. Participants will be offered reimbursement for all reasonable travel expenses in addition to a £25 online shopping voucher on completion of the study. If the participant requires respite for their relative with brain injury to enable them to participate, we will pay for the cost of attending their local Headway for one full or half day.

#### Collection of demographic data

Once informed consent has been given, participants will be asked to complete an online survey to report the following demographic data: age, gender, sexual orientation, ethnicity, first language, marital status, disability, religion and contact with NHS/third-sector organisations. These data will allow us to invite 20 participants to complete the ‘Life Threads’ approach, prioritising under-represented groups including LGBTQ+ (lesbian, gay, bisexual, transgender, and queer (or questioning)) communities, and Black, Asian and mixed ethnic groups. We will also ask the family member to provide data pertaining to the injured person to reflect the heterogeneity of the brain injury community and provide a contextual understanding of the nature and severity of brain injury. Data collected from those who are not invited to complete the ‘Life Threads’ approach will be fully anonymised and summarised as a comparator to the sample recruited.

#### Focus group 1: scene setting

Focus groups are a popular method in health services research for their ability to determine views and perspectives on healthcare interventions and initiatives.^61^ We will hold focus groups with approximately 4–6 participants in each in keeping with usual practice of 6–12^62^ or 4–8^63^ with smaller groups being better where participants need more time to share their experiences. Participants will be given the choice between attending focus groups in either face-to-face in person or online formats. Focus groups will last approximately 60–90 min.

In the first focus group (FG1), participants will be asked to introduce themselves and why they decided to join the study (see online supplemental material 1: focus group I schedule). From the responses to this question, we will determine the broader social context of each family system. Participants will then be introduced to the ‘Life Threads’ approach and invited to ask questions and seek clarification. In this study, the focus groups are primarily a means of data collection and, aside from introducing the materials, are not a specific part of the ‘Life Threads’ approach. However, we will need to carefully evaluate the additional value, if any, of sharing the ‘Life Threads’ with a wider group of family members.

#### Receipt of study materials

Having been introduced to the ‘Life Threads’ approach during FG1 the items depicted in figure 2 will be sent, in the post, to participants in this study together with further instructional text.

#### Self-directed time

Participants will be asked to engage with the ‘Life Threads’ approach using the study materials provided for approximately 1 month. Although ‘threads’ in the form of wool strands and small labels will be provided in the box, we will not limit the ways participants can use the materials and will suggest possibilities such as writing down meaningful events/experiences, adding photographs or using artefacts as representations of things that are meaningful to their story. Photographs and drawings are commonly used in arts-based health methods.^38^ Participants will be asked to think and reflect on these choices prior to their unstructured interview.

#### Unstructured individual interviews: articulating the story

We will use unstructured follow-up interviews to examine the individual experience, in a location chosen by the participants (see online supplemental material 2: interview schedule). An online interview may be conducted where requested or required. It is possible that engagement with the ‘Life Threads’ approach will vary from comprehensive and creative usage to no usage at all and if this arises, we will explore this variation in use. Participants who have engaged will be asked about their choices of artefacts/photographs and what they mean to them and their story. Participants who have not used the materials in their own time will be supported to work with the materials in a facilitated way with the researcher during the interview itself. These experiences may help us to understand the benefits of a more, or less, facilitated approach to using the ‘Life Threads’ approach. At the end of the individual interview, the researcher will take photographs of the participant’s creation and seek consent to share this with other participants in the second focus group.

#### Focus group 2: sharing the story

In the second FG (FG2), we will aim for participants to meet with the same family members from FG1 and we will share the photographs taken if consent is given (see online supplemental material 3: FG2 schedule). We will ask participants what worked well, what did not, what improvements could be made and if this would be helpful for others. We will talk about their experience of working with the materials on their own versus working with the materials with the researcher. We will ask what, if anything, they feel they have gained from the storytelling using the ‘Life Threads’ approach, over and above talking with us in the focus groups and individually. We will ask if they have continued to work with the materials since the interview or if they have shared them with anyone else outside of the research.

### Data analysis

This study will use ‘reflexive thematic analysis’ (rTA) to analyse both focus group and interview data involving six stages (see table 2).^64^,^68^ While we do have certain areas of interest, we will be open and curious about the data and prioritise an inductive approach to analysis. These analytical procedures will be applied within an interpretivist social constructivist orientation to focus on the narratives of participants as outlined in the Design section above.

**Table 2:** The six stages of reflexive thematic analysis^58^ including details of analytical activities for this study for each of the analytical tasks

Step	Analytical task	Analytical activities
1	Data familiarisation and writing familiarisation notes	Reading and re-reading data, listening to audio files, keeping a reflexive diary.
2	Systematic data coding	Independent coding of a sample of transcripts will be conducted by several members of the research team. Coding will be inductive, descriptive and latent. Coding decisions will be shared, and critically discussed before CJW proceeds to code full data set.
3	Generating initial themes from coded and collated data	Codes grouped and clustered, relationships and hierarchies examined by CJW supported by critical reflexive discussions with the research team.
4	Developing and reviewing themes	Research team will review the themes. PPI members will be consulted on the emerging themes to enhance depth and resonance of the analysis.
5	Refining, defining and naming themes	Finalising the analysis led by CJW with support from the research team. Research team will ensure each theme has a central organising concept and relationships between themes are well understood and articulated. PPI members will be consulted to ensure clarity.
6	Writing the report	Findings will be written up to answer the original research question and objectives. Vivid and compelling extracts of data will be chosen to support this.

PPIpatient and public involvement

We will identify what is common and what is particular and where engagement with the ‘Life Threads’ approach has worked well or failed to work as expected. We will consider participants’ descriptions of practical and experiential aspects of their use of the approach across the five questions, including but not limited to considerations of acceptability. Given this, we are not applying an a priori acceptability framework, although will consider such an approach to more focused evaluation of acceptability as part of a future feasibility RCT (see table 3 for how the analysis of data sources addresses the research objectives).

**Table 3:** How the analysis of data sources contributes to specific research objectives

	Analytical focus	Focus groups 1 and 2	Interviews
1	Explore if family members’ find storytelling through the ‘Life Threads’ approach useful as a strategy to support their individual subjective well-being and adjustment post-TBI.	x	x
2	Assess uncertainties in relation to the ‘Life Threads’ approach including acceptability; adherence; and level of facilitation required.	x	
3	Identify appropriate methods for a feasibility study including representative recruitment; choice of primary outcomes; mode of delivery and comparator arm(s).	x	
4	Understand how family members use the ‘Life Threads’ approach to understand the impact of TBI on themselves and their families.		x
5	Explore if the four domains of subjective experience post-TBI (displacing and anchoring; rupturing and stabilising; isolating and connecting; harming and healing) are representative of family member experiences.		x

TBItraumatic brain injury

Audio files will be sent to an approved third-party company for transcription and returned to the CI to be checked for accuracy and further anonymisation where required. Data collection and analysis will occur in parallel so that early analysis can inform later data collection if necessary. NVivo software will be used allowing researchers to organise the data, share coding decisions, discuss the generation of themes and confirm the origins of interpretation. Within relativist approaches, it is recognised that researcher objectivity is neither achievable nor sought after^69^ and in rTA the aim is to achieve ‘immersion, creativity, thoughtfulness and insight’.^68^ ^p.268^ Therefore, critical discussions with coresearchers and PPI members will facilitate greater engagement with the data and advance interpretation reached not check that the interpretation is ‘correct’.^68^

In keeping with the eight ‘Big Tent’ criteria proposed by Tracy^70^ and cognizant of the strategies for quality in rTA,^68^ we will seek sincerity of findings through reflexivity (reflexive diary and weekly research team meetings) and transparency of analytic decisions; credibility through inclusion of those with lived experience alongside qualitative research expertise within the research team; resonance through close engagement with the participants and the data generated and detailing of context to allow transferability; and rigour through collection of significant data over three time points and contexts from a relatively large sample for a study of this nature.

## Ethics and dissemination

### Ethical approval

The protocol described in this paper formed the basis of application for ethical approval (protocol version 3, dated 29 November 2023, which included non-substantial amendments to the protocol arising following ethics committee review). Approval has been given by the UK Health Research Authority and a favourable opinion provided by Nottingham 1 Research Ethics Committee (23/EM/0185, 4 September 2023). We will adhere closely to the Declaration of Helsinki and comply with the Data Protection Act^71^ and General Data Protection Regulations.^72^

The trial sponsor is Nottingham University Hospitals NHS Trust. Full data management procedures are described in the REC-approved protocol and comply with the information governance requirements of the University of Derby who are joint data controllers with Nottingham University Hospitals NHS Trust. Identifiable personal data will be stored away from anonymised research data and destroyed as soon as no longer needed. A password-protected data linkage file will be kept and this also deleted after the analysis has been completed.

### Informed consent

All participants will provide electronic informed consent. For all recruitment methods, interested parties will be able to contact the CI to request further details at which stage they will be sent a participant information sheet and example consent form. Family members can ask any questions they may have and receive answers to make an informed decision about participating in the study before providing verbal consent (see online supplemental material 4: informed consent form).

While we recognise that the injured and uninjured family member’s stories are intertwined, it is important to give the family member the right to choose themselves if they wish to participate. This requires balancing the wishes of the injured and non-injured family members. We, therefore, applied a procedure similar to one that was previously ethically approved and successfully applied to the recruitment of family members from NHS brain injury services.^73^ Accordingly, we will ask the prospective participants to share information about the study with their injured relatives. If the injured person wishes to talk to the researcher, we will ensure we make time to discuss the study with them and answer any questions they have. We will not seek formal consent from the injured person for their family members to take part. However, we will ask the family member to be open and honest with the injured person and encourage dialogue between the family members about the study. We will then ask the family member to confirm their relative does not object to their participation (where the injured person has the capacity to do so). If, despite this process, the family member indicates that their injured relatives do not agree with their participation, we will not proceed with recruitment of that participant.

### Assessment and management of risk

We recognise that distress is a normal and understandable response that can arise in the context of a family member sustaining a TBI. As a research team, we have clinical and research experience of responding to participant distress in this context and will respond sensitively and in a normalising way. At the same time, it is important we are conscious of potential mental health risk. Strategies to minimise the risk of harm to participants include being transparent about the potential for emotional distress, offering breaks during data collection, debriefing and signposting to organisations that can offer support including local Headway groups and self-referral to improving access to psychological therapies services. In cases of more severe distress, we will provide a 1-hour debrief with a psychologist. After this debrief, the psychologist can then refer to appropriate ongoing services if necessary. Debrief with a psychologist will be classed as an adverse event in this study. Serious adverse events will be reported to the study sponsor and ethics committee and recorded in keeping with Good Clinical Practice in Research guidance, in which all core research team members are trained.

### Dissemination

The study has been registered with the ISRCTN registry (www.isrctn.org, Trial ID: ISRCTN17392794, registered 22 December 2023) and adopted into the NIHR portfolio. On completion of this study, we will work with the PPI advisory group to develop an accessible summary of the results that will be shared with participants. Participants can also request an update on the study at any time prior to this. We will invite participants, PPI advisory group members and the wider research team to a local post-project dissemination event. We will return to Headway branches/groups and share our findings with staff and members and discuss the practice implications of using the ‘Life Threads’ approach. A summary of the study and its findings will be given to relevant stakeholders including NHS acute and community head injury services. We will continue our dialogue with our wider professional network and stakeholders to further develop these relationships and generate interest and support for our ongoing research. We will actively engage with social media sharing our results through an infographic on Twitter/X, LinkedIn and Facebook.

The study will be prepared and submitted for publication in an international open access journal and the study will be written up in accordance with established guidelines for reporting qualitative research and journal publisher and editor guidance as relevant.

## supplementary material

10.1136/bmjopen-2024-084204 online supplemental file 1

10.1136/bmjopen-2024-084204 online supplemental file 2

10.1136/bmjopen-2024-084204 online supplemental file 3

10.1136/bmjopen-2024-084204 online supplemental file 4
