# Supplementary material for: Developing the ‘Life Threads’ approach to support families after traumatic brain injury in UK community settings: protocol for a qualitative prefeasibility study
Source: BMJ Open. 2024 Oct 17;14(10):e084204. doi: 10.1136/bmjopen-2024-084204 (PMC11487829; doi:10.1136/bmjopen-2024-084204)
Supplement: online supplemental file 1 [file bmjopen-14-10-s001.pdf]

Focus group I schedule.

Pre-interview

A) (Establish Rapport) Hello everyone, thank you so much for agreeing to complete this focus group today. With me today is […] they will be watching the conversation and making a few notes. I just wanted to remind you that this discussion is confidential, and you will not be identifiable in any publications from the study. If at any time you would like to stop participating just let me know. Do you have any further questions? I will be recording the focus group on […insert device or devices…].

B) (Purpose & Motivation) Today, we would like to introduce you to the research team and to each other. We would then like to show you the 'Life Threads' approach and talk to you about what you will receive in the post and what we would like you to do before the next focus group. Please ask questions as we go. I expect the focus group to last about 60 – 90 minutes if it looks like we will go beyond this I will ask if you would like a break or if you are happy to continue. .

C) (Ground rules) I would just like to read out some ground rules and ask you to confirm you are happy to abide by these:

Ground rules: maintaining confidentiality, respect each other's views even where they differ from your own, allow space and time for people to contribute and try not talk over people.

You can leave the focus group at any time and a member of the research team will contact you to make sure you are ok. If we cannot reach you directly, we will call your emergency contact to ask them to contact you instead.

D) (Transition to interview) Okay, are you happy to get started? I will now turn on my recorder.

Start

1. Please would you introduce yourselves and why you decided to join the study.
2. Introduction to 'Life Threads' approach.
3. Invite questions and clarification.

Prepare to close
"Okay, if you are all comfortable and have asked all your questions, I will now turn the recording off.
Close:
"So that is the end of focus group I, you now have approximately one month before we meet again. I will send you an email with details of the individual interview and the second focus group. You should have my contact details if you have any questions. If you have said anything today you would prefer not to be used in this research you can ask for me not to report this, please just let me know within seven days. If anything from today has left you feeling like you need some additional support there are details of support services in the study information sheet"
