# Supplementary material for: Developing the ‘Life Threads’ approach to support families after traumatic brain injury in UK community settings: protocol for a qualitative prefeasibility study
Source: BMJ Open. 2024 Oct 17;14(10):e084204. doi: 10.1136/bmjopen-2024-084204 (PMC11487829; doi:10.1136/bmjopen-2024-084204)
Supplement: online supplemental file 2 [file bmjopen-14-10-s002.pdf]

Unstructured interview schedule

Pre-interview

A) (Establish Rapport) Hello, thank you so much for agreeing to complete this interview today. I just wanted to remind you that this interview is confidential, and you will not be identifiable in any publications from the study. If at any time you would like to stop the interview just let me know. Do you have any further questions?

B) (Purpose & Motivation) Today, I would like to ask you some questions about your use of the 'Life Threads' approach to understand the impact of traumatic brain injury on you. There are no 'right' answers, I am simply interested in your views and experiences. If there is a question you would prefer not to answer just let me know or if you do not understand a question, please ask me to clarify. I expect the interview to last about one hour but if it looks like we will go beyond this I will ask if you would like a break or if you are happy to continue. From this interview I'm hopefully going to understand more about your experiences of the way traumatic brain injury has affected you and your life.

C) (Transition to interview) Okay, are you happy to get started? I will now turn on my recorder.

Interview

A. Have you been able to work with the life thread materials that we sent you?

If yes - ask B
If no – ask C

B. Please tell me your story of how TBI has affected your life using your life thread creation.

C. Please tell me the story of how TBI has affected your life while working with the life thread materials in the box provided.

Praise

"what you said was very interesting thank you"
"It is very helpful that you explained it in that way"
"I really appreciate your candor and honesty, thank you"
"I can see that was a very difficult story to tell me so thank you for sharing it with me"

Probes

- And then what happened?
- When was that?
- When you said […] could you explain what you meant?
- You said […] why do you think that is?
- You spoke about […] could you tell me more about that?
- How did that make you feel?
- Could help me understand why it was you felt that way?
- Could give me an example so that I can understand this a bit more clearly?
- Why did you chose this [photograph/artefact]?

Prompts: Eye contact; leaning forward; open body language

Prepare to close
"Okay, I've asked all my questions now, thank you for everything you have told me we really appreciate you giving up your time to help us with this study. However, before we finish is there anything else you'd like to add that we might have missed?"

Close:
"I am now turning off the recording device"
"How do you feel now the interview is over?"

- Signpost interviewee to support services if necessary

"I would like to say a sincere thank you for helping us with this study we are enormously grateful to you.
What happens next is that we will transcribe the audio recording remove any personal details. Then we will then spend some time analysing your interview data and the data from other participants.
In the meantime, if you have any questions or queries, feel free to email me.
It was a pleasure to meet you, thank you again for helping us with this study".
