# Supplementary material for: Developing the ‘Life Threads’ approach to support families after traumatic brain injury in UK community settings: protocol for a qualitative prefeasibility study
Source: BMJ Open. 2024 Oct 17;14(10):e084204. doi: 10.1136/bmjopen-2024-084204 (PMC11487829; doi:10.1136/bmjopen-2024-084204)
Supplement: online supplemental file 3 [file bmjopen-14-10-s003.pdf]

Focus group II Schedule

Pre-interview

A) (Establish Rapport) Hello everyone, thank you so much for agreeing to come back and complete this focus group today. With me today is […] they will be watching the conversation and making a few notes. I just wanted to remind you that this discussion is confidential, and you will not be identifiable in any publications from the study. If at any time you would like to stop participating just let me know. Do you have any further questions? I will be recording the focus group on […insert device or devices…].

B) (Purpose & Motivation) Today, I'd like to ask you some questions about using the 'Life Threads' approach. There are no 'right' answers I am simply interested in your views and experiences. I will try not to contribute to the conversation, instead I will let you discuss the question in the group and I will take a few notes for follow-up questions later. If there is a question you would prefer not to answer it is fine not to contribute to the discussion. I expect the focus group to last about 60 – 90 minutes if it looks like we will go beyond this I will ask if you would like a break or if you are happy to continue. From this focus group I'm hopefully going to understand more about the usefulness of the 'Life Threads' approach for families after traumatic brain injury.

C) (Ground rules) I would just like to read out some ground rules and ask you to confirm you are happy to abide by these:

   Ground rules: maintain confidentiality, respect each other's views even where they differ from your own, allow space and time for people to contribute and try not talk over people.

   You can leave the focus group at any time and a member of the research team will contact you to make sure you are ok. If we cannot reach you directly, we will call your emergency contact to ask them to contact you instead.

D) (Transition to interview) Okay, are you happy to get started? I will now turn on my recorder.

Start

- In what did the 'Life Threads' approach helped or hindered you to understand the way TBI has affected your life?
- Are there any specific benefits you can identify from re-creating your story in this way?
- What problems did you have in using the 'Life Threads' approach?
- With whom and in what ways have you shared your story before?
- How does this experience of sharing your story in this study compare to those prior experiences?
- Have you shared your Life Thread creation with anyone else and if so what was your experience of this like?
- Were there any additional benefits from using the 'Life Threads' approach that would not have been possible if we had simply met to talk about your experiences?
- Have you found meeting in a group of any added value to meeting with a researcher individually?

Praise

"what you said was very interesting thank you"
"It is very helpful that you explained it in that way"
"I really appreciate your candor and honesty, thank you"
"I can see that was a very difficult story to tell me so thank you for sharing it with me"

Bringing in different participants into the conversation

- [insert name]… I noticed you were nodding, do you have a similar experience to [insert name]
- [insert name]… you seemed a little surprised by [insert name]'s account do you have a different view?
- [insert name] do you have a view on this?
- [insert name] would you like to share your experience

Bringing the conversation back in focus

- Thank you so much for sharing that story perhaps we could consider […insert focus of question…] more specifically now.

Probes

- Would anyone like to give me an example of […]?
- Does anyone have a [different/similar] experience?
- Can you think of any reason why you may have a [different/strong] views?
- Would anyone else like to talk about how they felt?
- Would anyone like to describe what happened to them?
- Does everyone agree that […]

Prompts: Eye contact; leaning forward; open body language

Prepare to close
"Okay, I've asked all my questions now, thank you for everything you have told me we really appreciate you giving up your time to help us with this study. However, before we finish is there anything else you'd like to add that we might have missed?"

Close:
"I am now turning off the recording device"
"How do you feel now the interview is over?"

- Signpost participants to support services if necessary

"I would like to say a sincere thank you for helping us with this study we are enormously grateful to you.
What happens next is that we will transcribe the audio recording remove any personal details. Then we will then spend some time analysing the focus group data and the data from other participants. We will write to you when we have the findings of the study and will also invite you to the dissemination event at the University of Derby should you wish to attend. These will most likely occur in the summer of 2024.
In the meantime, if you have any questions or queries, feel free to email me.
Thank you again for helping us with this study".
