# Supplementary material for: Developing the ‘Life Threads’ approach to support families after traumatic brain injury in UK community settings: protocol for a qualitative prefeasibility study
Source: BMJ Open. 2024 Oct 17;14(10):e084204. doi: 10.1136/bmjopen-2024-084204 (PMC11487829; doi:10.1136/bmjopen-2024-084204)
Supplement: online supplemental file 4 [file bmjopen-14-10-s004.pdf]

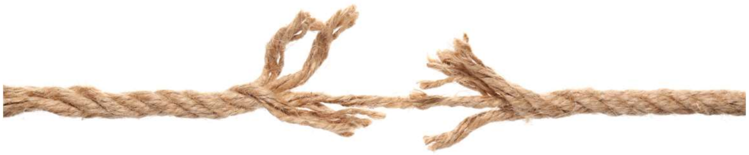

LIFE THREADS - TBI

Working with the 'Life Threads' approach to support families after traumatic brain injury.
IRAS Number: 329362
R&I reference number: 23NS011

Verbal Confirmation Script

Participant ID:	Initials:
Date of Birth:	
Principal Investigator: Dr Charlie Whiffin	
Date of verbal confirmation call: __/___/____ (dd/mmm/yyyy)<br><br>Time of verbal confirmation call: __/__ (00.00 hr)	
Format of verbal consent e.g. video call, phone call:	

Telephone/contact the participant and confirm that you are speaking to the relevant person, and that it is a convenient time to call (if not, establish if the participant wishes to be contacted at an alternative time; or whether the participant no longer wishes to be contacted regarding the study). Then continue with the script below:

Hello [*name of participant*], my name is Dr Charlie Whiffin and I am the lead Investigator working on the Life Threads - TBI study, which you have shown interest in.

You may have received some documents from us recently regarding the *Life Threads – TBI* research study and we wondered if you have had a chance to read them? ***(Allow participants to respond – if they have read it and no longer wish to be contacted with regards to this study, then thank them for their time and say goodbye – if they are unsure or more positive then continue below).***
I am calling today to invite you to take part in the focus groups and interview for the Life Threads – TBI study. We would like to find out if this way of supporting family members to make sense of what they have been through after the traumatic brain injury of a family member is useful. To do this we would like to invite you to join two focus groups, work with the Life Threads approach and to have an individual interview.

The Ethics Committee, whose role it is to scrutinise research and protect patients, has agreed that we can obtain verbal confirmation from you over the phone, but please let me reassure you that whatever you decide, it will not change your rights in anyway. We will then send this document to you via DocuSign for you to sign, as evidence of your informed consent. Do you have any questions you would like to ask me at this stage?

Record participant's response: Yes / No
If yes, record any questions and responses given, below:

Just so that I can check that I have explained myself clearly to you, can you please confirm if you understand what I have told you?

Record participant's response: Yes / No
If you are happy with my responses, can you please let me know whether you agree or not, to take part in the Life Threads - TBI study?

Record participant's response: Yes / No
(If participant answered yes, continue overleaf; if no, then thank the participant for their time and say goodbye)
Finally, could I please ask you to confirm a few details to record your confirmation?

Version and date (dd/mmm/yyyy) of information sheet read by participant:
Version: ____ Dated: ___ / ____ / _____

Date the study information was received (dd/mmm/yyyy)? ___ / ____ / _____

Address:………
Email: ………

We will send this form to you via DocuSign. Once you have provided an electronic signature you will be able to download a copy for your records.

Please would you confirm the name and address of your GP so we can let them know you are participating in this study.
Address:………
Email: ………

In addition, please would you identify an emergency contact who we can call if during the study we cannot reach you directly.
Address:………
Email: ………

PARTICIPANT VERBAL CONFIRMATION FORM
IRAS Number: 329362
R&I reference number: 23NS011

Read out the below clauses one at a time and confirm the participant agrees with each of them

		Please initial (researcher)
1	After explaining the study information to the participant, they have confirmed that they understand and agree to participate in the above research study. The participant confirms they have had the opportunity to ask questions and have these answered satisfactorily	***Yes*** ***No***
2	The participant confirms that the family member who sustained a traumatic brain injury does not object to their participation in this study.	***Yes*** ***No***
3	The participant understands that the study is voluntary, and that they can withdraw at any time without their medical care or legal rights being affected.	***Yes*** ***No***
4	The participant understands that should they withdraw, they can only request interview data be withdrawn from the study up to seven days after, and that focus group data can NOT be withdrawn; however they can request their data are not included in publications arising from this study up to seven days after.	***Yes*** ***No***
5	The participant understands that the verbal information they provide will be recorded on an encrypted device and that interviews held online will be video recorded.	***Yes*** ***No***
6	The participant understand that anonymised quotes will be shared in publications arising from this study and that they will not be identified in these. They also understand that despite anonymisation that people close to them may still be able to recognise the information they have provided due to unique events and experiences they describe.	***Yes*** ***No***
7	The participant understands that the information collected about them will be used to support other research in the future, and may be shared anonymously with other researchers.	***Yes*** ***No***
8	The participant agrees that their GP will be notified of their participation in this study.	***Yes*** ***No***
9	The participant understand that data collected during the study, may be looked at by individuals from Nottingham University Hospitals Trust where it is relevant to them taking part in this research. The participant gives permission for these individuals to have access to their study records.	***Yes*** ***No***
10	The participant understands that after providing their details to the research team they may, or may not, be invited to participate further. If they are not invited to continue, they understand that details they have provided will still be securely archived by the Nottingham University Hospitals NHS Trust for a minimum of five years when arrangements for confidential destruction will then be made.	***Yes*** ***No***
11	The participant would like to be informed about future research studies.	***Yes*** ***No***
12	The participant agrees to take part in the study.	***Yes*** ***No***

Participant

Full name (*block capitals*):

Date of Birth: DD/MMM/YYYY ………. / ……….... / ………..…

Date/time verbal confirmation provided by participant: DD/MMM/YYYY Time (24 hr) ………. / ……….... / ………..…

Person taking confirmation
I have explained the study to the above named participant and they have indicated their willingness to participate.
Full name (*block capitals*):

Signature:

Date: DD/MMM/YYYY ………. / ……….... / ………..…

To be completed via DocuSign

I give my consent to participate in the study as described above.

______________________	____________	______________________
Name of the participant *(Print)*	date	Participant's signature

Please retain download a copy of this document for your own records.

Original to be stored in site file. One copy to be retained by the participant.
